# NO-Donating Aspirin and Aspirin Partially Inhibit Age-Related Atherosclerosis but Not Radiation-Induced Atherosclerosis in ApoE Null Mice

**DOI:** 10.1371/journal.pone.0012874

**Published:** 2010-09-21

**Authors:** Saske Hoving, Sylvia Heeneman, Marion J. J. Gijbels, Johannes A. M. te Poele, Manlio Bolla, Jeffrey F. C. Pol, Michelle Y. Simons, Nicola S. Russell, Mat J. Daemen, Fiona A. Stewart

**Affiliations:** 1 Division of Experimental Therapy, The Netherlands Cancer Institute, Amsterdam, The Netherlands; 2 Division of Radiotherapy, The Netherlands Cancer Institute, Amsterdam, The Netherlands; 3 Department of Pathology, University of Maastricht, Cardiovascular Research Institute Maastricht, Maastricht, The Netherlands; 4 Department of Molecular Genetics, University of Maastricht, Cardiovascular Research Institute Maastricht, Maastricht, The Netherlands; 5 NicOx SA, Sophia-Antipolis, France; Universität Würzburg, Germany

## Abstract

**Background:**

We previously showed that irradiation to the carotid arteries of ApoE^−/−^ mice accelerated the development of macrophage-rich, inflammatory atherosclerotic lesions, prone to intra-plaque hemorrhage. In this study we investigated the potential of anti-inflammatory and anti-coagulant intervention strategies to inhibit age-related and radiation-induced atherosclerosis.

**Methodology/Principal Findings:**

ApoE^−/−^ mice were given 0 or 14 Gy to the neck and the carotid arteries and aortic arches were harvested at 4 or 30 weeks after irradiation. Nitric oxide releasing aspirin (NCX 4016, 60 mg/kg/day) or aspirin (ASA, 30 or 300 mg/kg/day) were given continuously in the chow. High dose ASA effectively blocked platelet aggregation, while the low dose ASA or NCX 4016 had no significant effect on platelet aggregation. High dose ASA, but not NCX 4016, inhibited endothelial cell expression of VCAM-1 and thrombomodulin in the carotid arteries at 4 weeks after irradiation; eNOS and ICAM-1 levels were unchanged. After 30 weeks of follow-up, NCX 4016 significantly reduced the total number of lesions and the number of initial macrophage-rich lesions in the carotid arteries of unirradiated mice, but these effects were not seen in the brachiocephalic artery of the aortic arch (BCA). In contrast, high dose ASA lead to a decrease in the number of initial lesions in the BCA, but not in the carotid artery. Both high dose ASA and NCX 4016 reduced the collagen content of advanced lesions and increased the total plaque burden in the BCA of unirradiated mice. At 30 weeks after irradiation, neither NCX 4016 nor ASA significantly influenced the number or distribution of lesions, but high dose ASA lead to formation of collagen-rich “stable” advanced lesions in carotid arteries. The total plaque area of the irradiated BCA was increased after ASA, but the plaque burden was very low compared with the carotid artery.

**Conclusions/Significance:**

The development and characteristics of radiation-induced atherosclerosis varied between different arteries but could not be circumvented by anti-inflammatory and anti-coagulant therapies. This implicates other underlying mechanistic pathways compared to age-related atherosclerosis.

## Introduction

The long-term survival of cancer patients is improving due to earlier diagnosis and better treatment. However, this results in an increased number of patients at risk for developing late side effects. In 2002, the total number of recorded cancer survivors in the world was estimated to be just under 25 million and in 2025 this number will approach 50 million [1]. At least half of these patients will have received radiotherapy. Early and late side effects limit radiation dose and have significant impact in terms of both quality of life and overall survival. Whereas typical side effects are systemic in the case of drug therapies, they are local or loco-regional in irradiated patients. Cardiovascular and cerebrovascular diseases are known complications of radiotherapy for many types of cancer, including head and neck, breast and Hodgkin's lymphoma [2]–[4]. There is good evidence to identify radiation as an independent risk factor in human vascular disease, in addition to the classical risk factors like hypercholesterolemia, age, diabetes, hypertension, smoking, stress and lack of exercise. Several studies have shown that irradiation of the carotid arteries leads to development of atherosclerosis, which increases the risk of vascular stenosis, thromboembolism and stroke [2], [5], [6].

We have shown in previous studies that both fractionated irradiation and high single dose irradiation to the carotid arteries accelerate the development of atherosclerosis in apolipoprotein E-deficient (ApoE^−/−^) mice and predispose to the formation of an inflammatory, prothrombotic plaque phenotype [7], [8]. Other experimental studies in hypercholesterolemic rabbits or mice fed high fat diets have also demonstrated that single dose irradiation of large arteries results in an accelerated formation of atherosclerotic plaques [9], [10].

Inflammation has been shown to both initiate and maintain the formation of atherosclerosis in coronary and peripheral arteries. One of the earliest events in the development of atherosclerotic lesions is the accumulation of monocytes/macrophages and lymphocytes in the intima of large vessels, which is mediated by distinct endothelial cell adhesion molecules like Intracellular Adhesion Molecule-1 (ICAM-1), Vascular Cell Adhesion Molecule-1 (VCAM-1) and selectins [11], [12]. Platelets play an important role in vascular inflammation and initiation of atherosclerosis, through release of their own pro-inflammatory mediators and close interaction with other cell types (endothelial cells, leukocytes and smooth muscle cells) [13].

Acetylsalicylic acid (ASA), an anti-platelet drug, is recommended and prescribed in the prevention of vascular events in a variety of clinical conditions, such as myocardial infarction, thrombosis and stroke [14], [15]. Its beneficial effect is not only by blocking platelets' ability to aggregate, but also by other mechanisms, such as preventing thromboxane A2 (TxA2)-induced vasoconstriction and reducing inflammation. A major limitation to long term ASA therapy is the risk of gastrointestinal events such as ulceration and bleeding due to the reduced mucosal production of COX-1 derived cytoprotective prostaglandins [16]. An approach to reduce the gastric damaging effects of ASA is to create a new molecule in which the acetylsalicylic moiety is chemically linked to a nitric oxide (NO)-donating group. The compound used in this study is NCX 4016 (previously called NO-ASA) that has been shown to possess at least the same anti-inflammatory activity as ASA, but with a sparing action on the gastric mucosa of animals and humans [17], [18]. In addition, NCX 4016 has been shown to be more effective than ASA in preventing neointimal remodeling and reducing inflammatory cell invasion and stenosis after arterial injury in hypercholesterolemic mice [19], [20].

Since inflammatory and thrombotic events are dominant features in radiation-induced atherosclerosis, we hypothesized that anti-inflammatory and anti-thrombotic agents should prevent or ameliorate radiation-induced atherosclerosis. Therefore, we examined the effects of ASA and NCX 4016 on the development of radiation-induced atherosclerosis and compared this with age-related atherosclerosis in ApoE^−/−^ mice.

## Results

### Evaluation of systemic effects

We observed an increase in body weight during the 30 week follow-up period in all mice, but irradiated mice gained less weight than unirradiated mice. In the unirradiated group, mice given NCX 4016 or ASA containing chow gained less weight than mice given control chow, but this difference was not seen in the irradiated mice (Table 1). No significant differences in food intake between treatment groups were found (Table 1). The measured food intake is a slight overestimation as spillage was not measured. At the end of the study all treatment groups had comparable total cholesterol levels. Small differences were seen between groups in HDL and triglyceride levels, but this seems not to be due to irradiation or treatment with NCX 4016 or ASA (Table 1).

**Table 1:** Estimated food/drug intake, body weight and plasma cholesterol levels.

Treatment	Food intake	Drug intake	Final weight	Cholesterol (mmol/L)			
	(g/mouse/day)	(mg/kg/day)	(% of initial weight)	Total	HDL	LDL	Triglycerides
*0 Gy*							
Control chow (n = 9)	4.7±0.7	-	152±2	13.5±1.2	5.4±0.3	7.1±1.1	1.8±0.3
NCX 4016 chow (n = 12)	4.7±0.4	86±2	141±3 #	15.0±0.3	5.2±0.1	9.0±0.2	1.7±0.2
ASA high chow (n = 12)	4.5±0.8	457±16	118±1 #	13.7±1.2	4.4±0.2 #	8.6±0.9	1.6±0.1
*14 Gy*							
Control chow (n = 10)	4.2±0.5	-	122±3 *	12.6±1.2	4.4±0.2	7.4±0.9	1.7±0.2
NCX 4016 chow (n = 11)	4.4±0.5	82±3	126±3 *	13.7±1.2	4.6±0.2 *	8.5±0.9	1.5±0.3
ASA high chow (n = 8)	4.1±0.9	427±18	123±1 *	14.2±1.2	5.0±0.2 #	8.6±1.1	1.1±0.1 # *
ASA low chow (n = 11)	4.9±0.8	49±2	127±2	12.5±0.5	3.9±0.1	8.1±0.4	1.2±0.1 #

Values are group means ± SEM. Cholesterol levels were measured in half of the mice after 30 weeks of treatment.

*p<0.05 compared with unirradiated mice with the same chow.

# p<0.05 compared with control chow with the same radiation dose.

**Figure 1:**
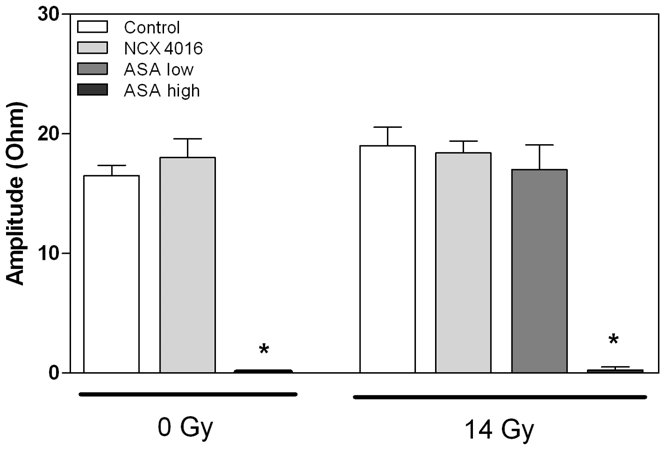
Platelet aggregation ex vivo in whole blood of unirradiated and irradiated mice treated with control chow, NCX 4016 (60 mg/kg/day), low dose (30 mg/kg/day) or high dose ASA containing chow (300 mg/kg/day). Data represents group means ± SEM (n = 4–6) * Indicates significant differences between high dose ASA and control chow (p<0.05 by Mann-Whitney U test).

There was an almost complete inhibition of platelet aggregation, measured ex vivo after triggering with arachidonic acid in mice treated with high dose ASA. Treatment with NCX 4016 or low dose ASA did not inhibit platelet aggregation and irradiation had no influence on platelet aggregation (Figure 1).

### Effect of NCX 4016 and ASA on age-related atherosclerosis

**Figure 2:**
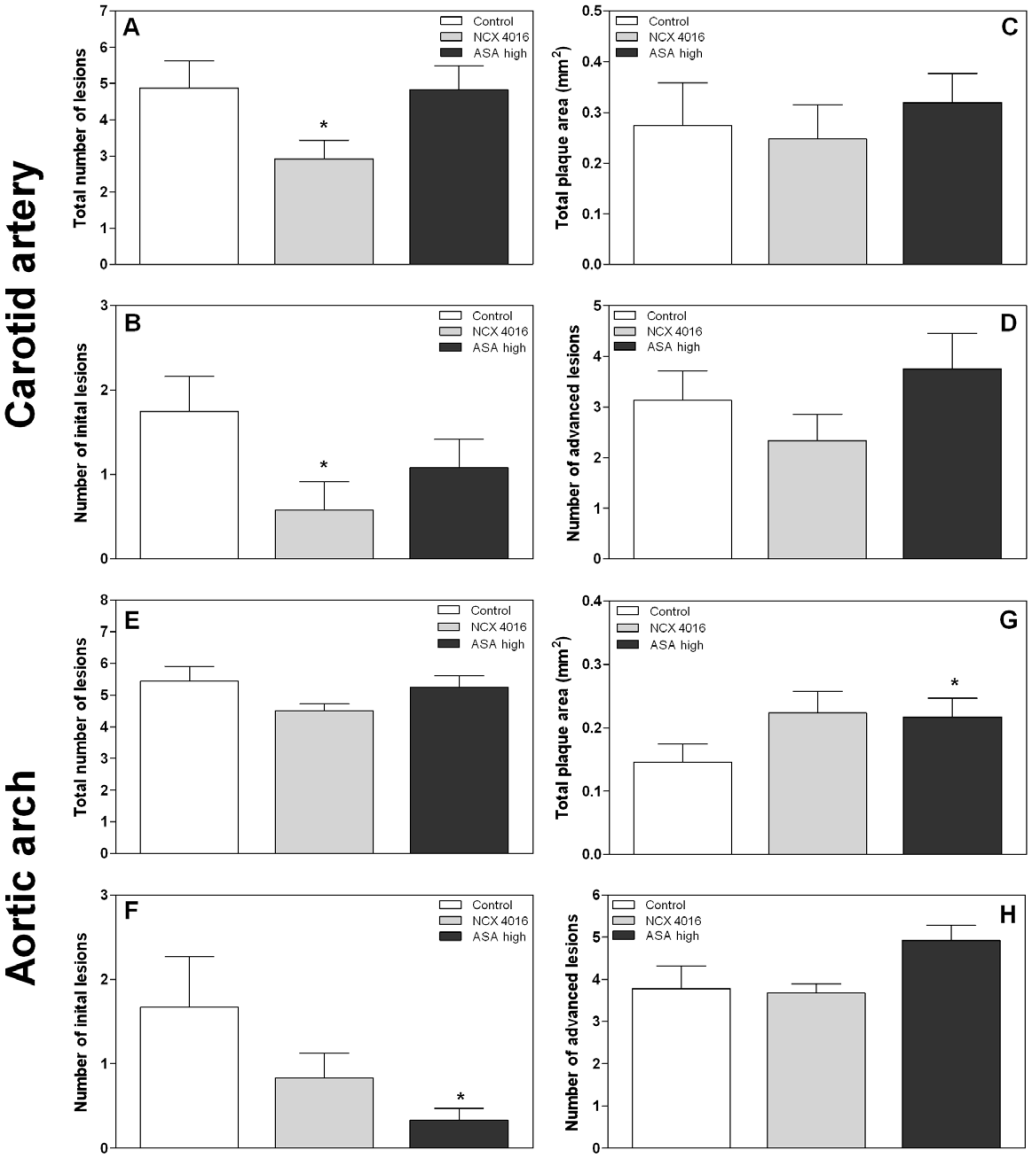
Morphometric analysis of lesions in unirradiated carotid arteries (A–D) and aortic arches (E-H) of ApoE^−/−^ mice treated with control chow (n = 9), NCX 4016 (60 mg/kg/day) (n = 12) or high dose ASA containing chow (300 mg/kg/day) (n = 12) for 30 weeks. Bar graphs show the mean number of total (A, E), initial (macrophage rich, without a thick fibrous cap) (B, F) and advanced (well-defined, necrotic/lipid core or thick fibrous cap) (D, H) lesions per animal. Total plaque area is shown in C and G. Data represents group means ± SEM. * Indicates significant differences between NCX 4016 or high dose ASA and control chow (p<0.05 by Mann-Whitney U test).

To examine whether NCX 4016 and high dose ASA were able to reduce age-related atherosclerosis, mice were sacrificed 30 weeks after start of the treatment and the number of lesions and the total plaque area in both carotid arteries or the brachiocephalic artery of the aortic arch (BCA) were determined. All mice developed atherosclerosis with age. Treatment with NCX 4016 resulted in a significant decrease in the total number of lesions (p = 0.04) and number of initial lesions (p = 0.02) in carotid arteries compared with the control mice, while high dose ASA had no effect on these lesions (Figure 2A, 2B). The mean size of individual initial lesions in the carotid artery was not reduced in the NCX 4016 treated mice compared with the control mice (0.013±0.009 versus 0.010±0.008 mm^2^), while high dose ASA caused an increased individual initial plaque size (0.025±0.012 mm^2^; p = 0.001). Neither NCX 4016 nor ASA resulted in a decreased number or mean individual size of advanced lesions, or a decreased total plaque burden of the carotid artery (Figure 2C and D).

In the unirradiated BCA of the aortic arch there was a trend towards a decreased total number of lesions in the NCX 4016 treated mice compared to the control mice (p = 0.052) (Figure 2E), but no significant decrease in the number of initial lesions (Figure 2F), or total plaque burden (Figure 2G). High dose ASA caused a significant decrease in the number of initial lesions in the BCA, which was not seen in the carotid artery. However, the total plaque area was increased after high dose ASA (p = 0.028) (Figure 2G). This increase in plaque area was due to an increased mean size of individual advanced lesions (0.172±0.078 and 0.233±0.092 mm^2^ in control and high dose ASA, respectively).

**Figure 3:**
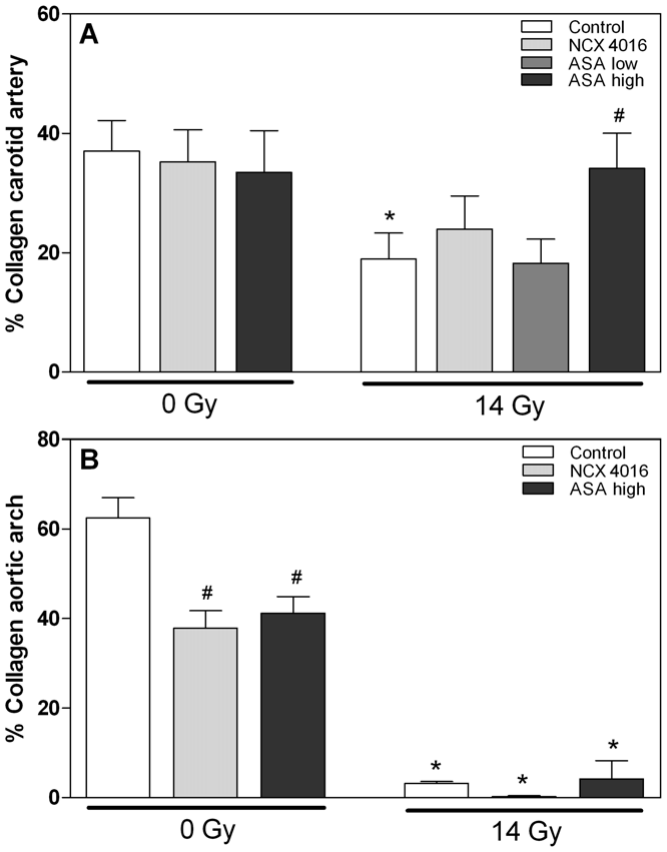
Collagen content of advanced lesions in carotid arteries (A) and aortic arches (brachiocephalic artery) (B) of unirradiated and irradiated male ApoE−/− mice treated with control chow, NCX 4016 (60 mg/kg/day), low dose (30 mg/kg/day) or high dose ASA (300 mg/kg/day) containing chow for 30 weeks. Data represents group means ± SEM. * p<0.05 compared with unirradiated mice with the same chow, *^#^* p<0.05 compared with control chow (Mann-Whitney U test).

Plaques in the carotid arteries were scored for the presence of granulocytes, erythrocyte/iron-containing macrophages and fibrin deposits. Neither NCX 4016 nor high dose ASA caused phenotypic changes compared to the control mice (Table 2). Furthermore, these drugs had no influence on the collagen content of advanced lesions (Figure 3A). On the other hand, both NCX 4016 and high dose ASA decreased the collagen content of advanced BCA lesions, although the percentage collagen is still quite high (Figure 3B).

**Table 2:** Semi-quantitative analysis of thrombotic features in unirradiated and irradiated carotid arteries of ApoE^−/−^ mice treated with control chow, NCX 4016 (60 mg/kg/day), low dose ASA (30 mg/kg/day) or high dose ASA (300 mg/kg/day) for 30 weeks.

Treatment	Granulocytes	Fe/Macr, Ery/Macr	Fibrin
*0 Gy*			
Control chow	4/9	4/9	0/9
NCX 4016 chow	2/9	4/9	2/9
ASA high chow	8/12	5/12	1/12
*14 Gy*			
Control chow	8/10	10/10*	6/10*
NCX 4016 chow	6/11	9/11	6/11
ASA high chow	6/8	6/8	3/8
ASA low chow	11/11	10/11	5/11

*p<0.05 compared with unirradiated mice with the same chow.

In conclusion, NCX 4016 significantly reduced the total number of lesions and the number of initial lesions in the carotid arteries of unirradiated mice, but was not able to reduce the total plaque burden. High dose ASA reduced the number of initial lesions, but increased the plaque area of BCA lesions in unirradiated mice.

### Effect of NCX 4016 and ASA on inflammatory and thrombotic endothelial markers 4 weeks after irradiation

**Figure 4:**
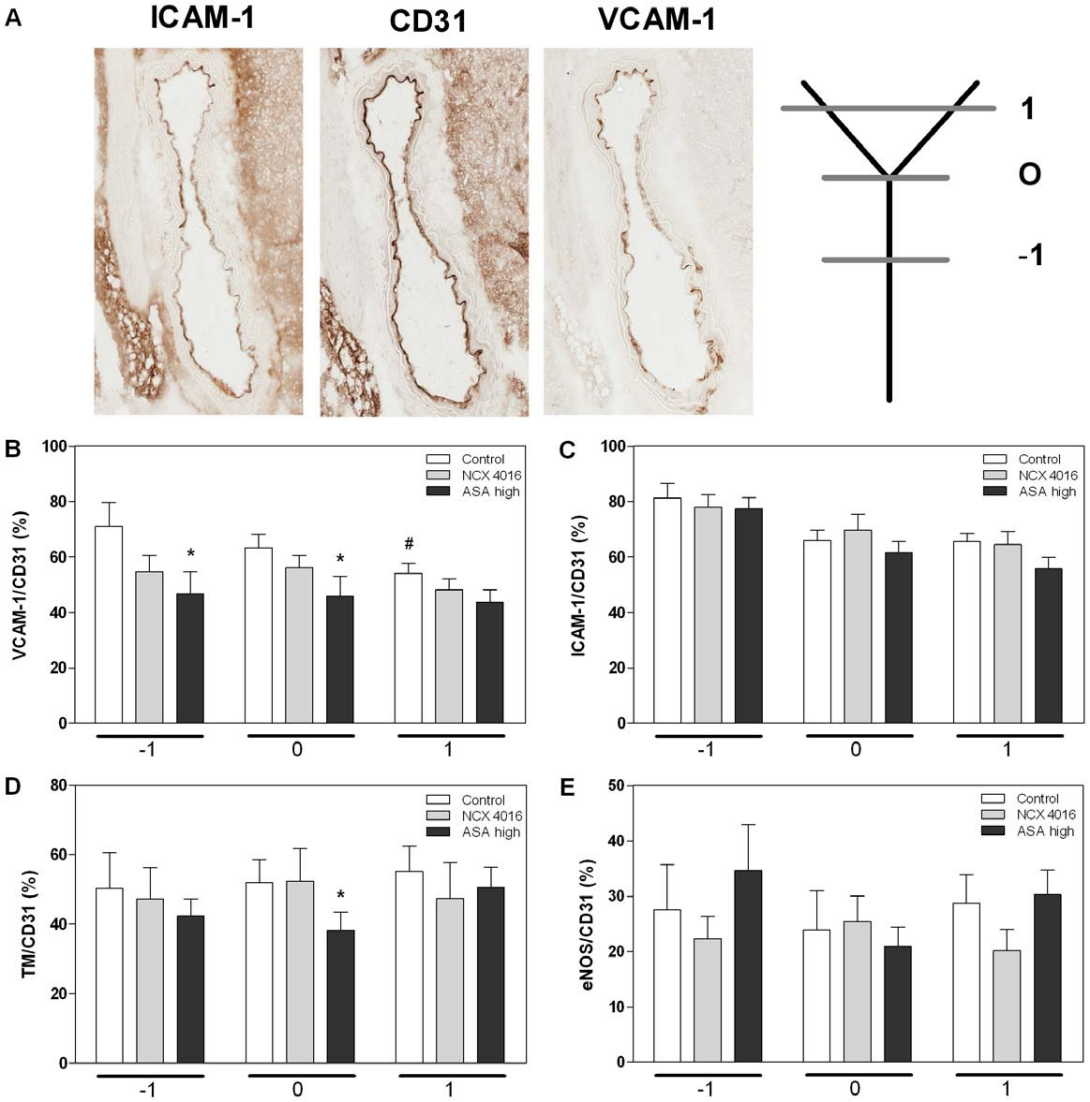
Immunohistochemical stainings of inflammatory and thrombotic markers. A) VCAM-1 and ICAM-1 expression of carotid arteries with adjacent CD31 staining. Expression levels of VCAM-1 (B), ICAM-1 (C), thrombomodulin (D) and eNOS (E) in endothelial cells of carotid arteries of irradiated ApoE^−/−^ treated with control chow, NCX 4016 (60 mg/kg/day) or high dose ASA (300 mg/kg/day) containing chow. Bar graphs show the mean expression levels at three positions in the carotid artery: −1; below the bifurcation, 0; at the bifurcation, 1; above the bifurcation and are corrected for the percentage of intact endothelium detected with CD31. Data represents group means ± SEM (n = 10) * Indicates significant differences between high dose ASA and control chow at the same position and ^#^ indicates significant difference between position -1 and 1(p<0.05 by Mann-Whitney U test).

We examined the carotid arteries of ApoE^−/−^ mice sacrificed at 4 weeks after the start of the irradiation (14 Gy) for the presence of fatty streaks. In 3 out of 10 mice that received control chow small fatty streaks were found, while in the mice treated with NCX 4016 or high dose ASA only 1 out of 10 mice developed small fatty streaks (not significant). To investigate the effect of NCX 4016 and ASA on the vascular, inflammatory and thrombotic response associated with atherosclerosis, we performed quantitative immunohistochemical stainings on carotid arteries at different locations (below, at and above the bifurcation, Figure 4A). First, a CD31 staining was performed to determine the percentage of intact endothelium. This was 88% in the control group, 94% in the NCX 4016 group (p =  0.013 compared with control) and 89% in the high dose ASA group. The percentage of endothelial cells expressing VCAM-1 was significantly lower above the bifurcation compared with below the bifurcation (p = 0.019) (Figure 4B) and there was a trend for decreased ICAM-1 expression around and above the bifurcation compared with below the bifurcation (p = 0.06 and p = 0.07, respectively) (Figure 4C). This indicates that there is site-specificity for expression of these proteins after irradiation. Neither thrombomodulin nor eNOS showed different expression levels at the three positions (Figure 4D and E).

Next, we tested the effect of NCX 4016 and high dose ASA on the expression levels of these molecules. Treatment with high dose ASA resulted in a decreased VCAM-1 expression below and at the bifurcation (p = 0.031 and p = 0.05, respectively) compared with mice given control chow. In addition to the reduction in percentage of endothelial cells expressing VCAM-1, there was a reduction in the intensity of positive immunostaining below the bifurcation (p = 0.034) (Table 3). There was no effect of the drugs on the percentage of endothelial cells expressing ICAM-1 (Figure 4C). However, a slightly increased intensity of positive immunostaining was seen at the bifurcation of carotid arteries of mice treated with NCX 4016 compared with control mice (p = 0.017) (Table 3). High dose ASA resulted in a decreased expression of thrombomodulin at the bifurcation compared with control mice (p = 0.041) (Figure 4D) and also a decreased intensity of positive immunostaining was seen at the same position (p = 0.041) (Table 3). Neither NCX 4016 nor high dose ASA had an effect on the expression levels of eNOS (Figure 4E).

**Table 3:** Intensity data of VCAM-1, ICAM-1, thrombomodulin and eNOS quantitative immunohistochemical stainings in carotid arteries of irradiated ApoE^−/−^ mice treated with control chow, NCX 4016 (60 mg/kg/day) or high dose ASA (300 mg/kg/day) containing chow for 4 weeks.

Treatment	Position	Intensity			
		VCAM-1	ICAM-1	TM	eNOS
Control chow	−1	194±3	200±2	174±4	205±3
	0	194±3	202±1	173±3	207±3
	1	198±2	203±1	171±4	204±2
NCX 4016 chow	−1	201±3	196±2	184±5	206±2
	0	198±2	198±2 *	180±6	205±2
	1	202±2	201±1	183±5	207±2
ASA high chow	−1	203±3 *	198±2	181±4	202±2
	0	201±3	202±2	183±4 *	205±2
	1	204±2	205±2	181±5	203±2

Average ± SEM (n = 10).

*compared with control chow at the same position (p<0.05).

### Effect of NCX 4016 and ASA on radiation-induced atherosclerosis

**Figure 5:**
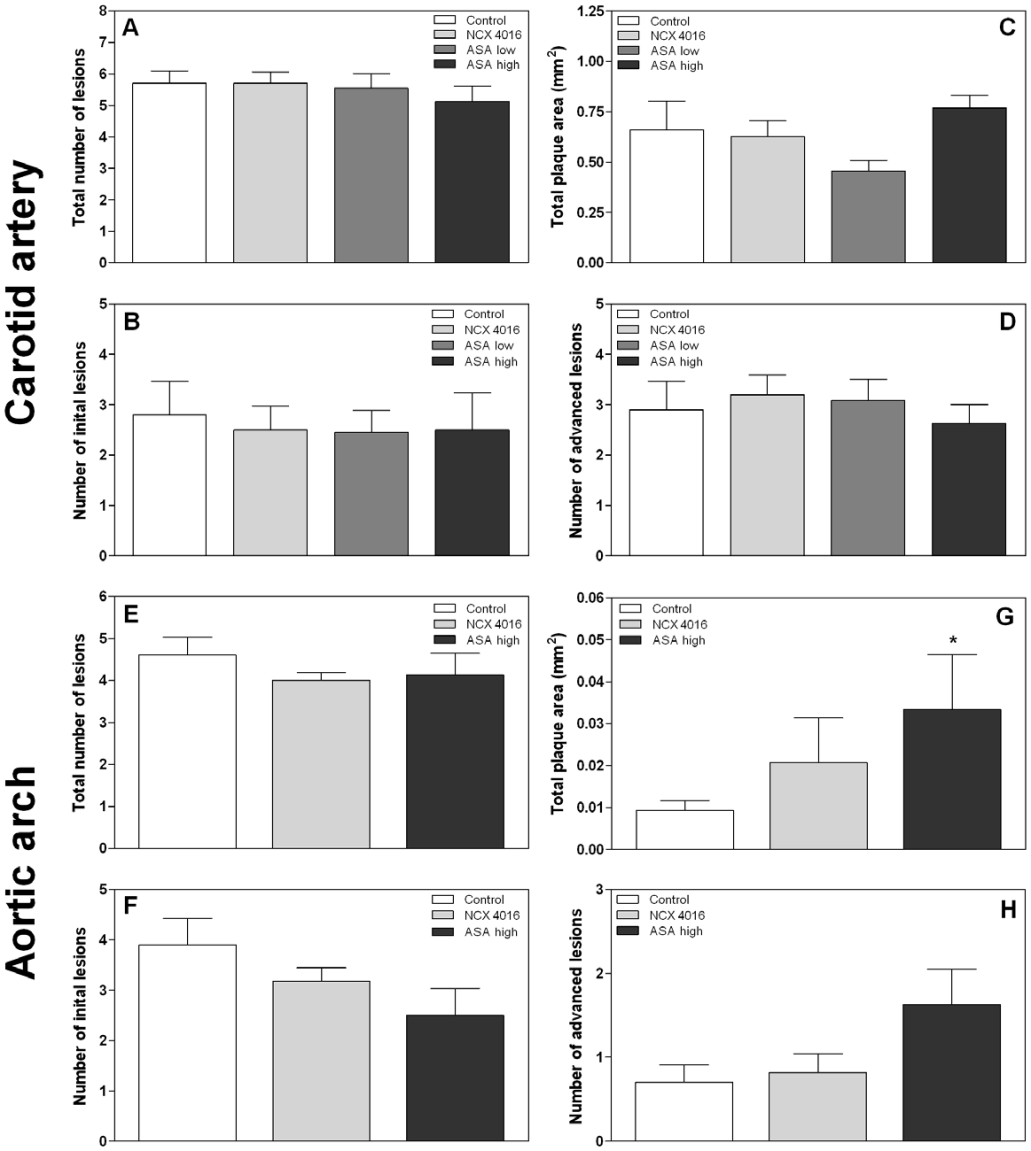
Morphometric analysis of lesions in irradiated carotid arteries (A–D) and aortic arches (E-H) of ApoE^−/−^ mice treated with control chow (n = 10), NCX 4016 (60 mg/kg/day) (n = 11), low dose (30 mg/kg/day) (n = 11) or high dose ASA containing chow (300 mg/kg/day) (n = 8) for 30 weeks. Bar graphs show the mean number of total (A, E), initial (macrophage rich, without a thick fibrous cap) (B, F) and advanced (well-defined, necrotic/lipid core or thick fibrous cap) (D, H) lesions per animal. Total plaque area is shown in C and G. Data represents group means ± SEM. * Indicates significant differences between high dose ASA and control chow (p<0.05 by Mann-Whitney U test).

We next tested whether NCX 4016 or ASA was able to reduce radiation-induced atherosclerosis. Neither NCX 4016 nor ASA significantly reduced the total number of lesions or the distribution of lesions in the carotid arteries 30 weeks after start of the treatment (Figure 5A/B/D). The drugs also had no effect on the mean size of individual initial lesions (0.071±0.040 mm^2^ in control), although low dose ASA significantly decreased the mean size of individual advanced lesions in the carotid artery (0.091±0.070 versus 0.156±0.113 mm^2^). In the BCA, neither NCX 4016 nor high dose ASA influenced the number or distribution of lesions but the total plaque area was significantly increased in the ASA group (Figure 5G). However, all lesions in these irradiated BCA arteries were very small and no differences were seen in the mean size of individual initial (0.008±0.005, 0.012±0.012 and 0.016±0.006 in control, NCX 4016 and high dose ASA, respectively) or advanced lesions (0.014±0.015, 0.059±0.072 and 0.051±0.048 in control, NCX 4016 and high dose ASA, respectively) The total plaque areas were 10-fold less than in the corresponding unirradiated arteries (Figure 2G).

All carotid lesions of irradiated mice given control chow showed erythrocyte/iron-containing macrophages compared with 44% of the unirradiated mice s (p = 0.01) (Table 2). Fibrin deposits were not seen in lesions of the unirradiated control mice, but 60% of the irradiated mice had fibrin deposits in the lesions (p = 0.01). Neither NCX 4016 nor ASA were able to reverse the hemorrhagic phenotype of the irradiated lesions (Table 2).

Analyses of collagen content of the advanced carotid lesions showed a decrease in collagen content of irradiated mice compared with lesions of unirradiated mice receiving control chow (19% versus 37%; p = 0.009) (Figure 3A). This is consistent with our previous findings and indicates reduced plaque stability in the irradiated lesions [7], [8]. High dose ASA led to a 1.8-fold increased collagen content compared to control chow (p = 0.045), while low-dose ASA and NCX 4016 had no effect on the collagen content of irradiated lesions (Figure 3A). The effect of irradiation on collagen content was more pronounced in the advanced BCA lesions (3% versus 62% in irradiated and unirradiated lesions; p = <0.0001). High dose ASA and NCX 4016 were not able to enhance collagen content of the aortic arch lesions compared with control chow (Figure 3B).

## Discussion

In previous studies we have shown that irradiation of the carotid arteries of ApoE^−/−^ mice accelerated the development of atherosclerosis and predisposed to the formation of an inflammatory, thrombotic plaque phenotype. In the present study we tested whether anti-inflammatory and anti-thrombotic agents like ASA and NCX 4016 were able to inhibit the development of radiation-induced atherosclerosis or to reverse the inflammatory plaque phenotype. NCX 4016 significantly reduced the total number of atherosclerotic lesions and the number of initial macrophage-rich lesions in carotid arteries of unirradiated mice, but these effects were not maintained in irradiated mice. ASA did not reduce lesion number or size in carotid arteries but did lead to the formation of collagen-rich stable plaque phenotype in irradiated mice. High dose ASA reduced the number of initial lesions in the unirradiated aortic arch, but once again the effect was not maintained in the irradiated mice. High dose ASA caused an increased plaque area of the BCA lesions in the aortic arch and did not lead to a more collagen-rich plaque phenotype in irradiated mice.

### Anti-inflammatory drugs and age-related atherosclerosis

Several pharmacological therapies have been designed to reduce the development and progression of age-related atherosclerosis. ASA, an anti-platelet drug, is used in the prevention of cardiovascular events in a variety of clinical conditions, such as myocardial infarction, thrombosis and stroke [14], [15]. Its beneficial effect is not only by blocking platelets' ability to aggregate, but also reducing inflammation. In the present study we focused on ASA and NCX 4016, since these drugs have both anti-inflammatory and anti-thrombotic activities.

NCX 4016, but not ASA, inhibited the initiation of atherosclerotic lesions in carotid arteries of unirradiated ApoE^−/−^ mice. There were significantly fewer total lesions compared with age-matched controls and a marked reduction in the initial lesions without a fibrous cap. However, this did not result in a reduced total plaque area. In hypercholesterolemic LDLR^−/−^ mice, treatment with NCX 4016 significantly reduced atherosclerotic lesion area in the carotid artery after 12 weeks of treatment, while ASA had no effect on the lesion size [21]. In an injury-induced neointima formation model it was also found that NCX 4016, but not ASA, reduced neointimal expansion [20], [22] and this reduction was associated with a marked deficit in inflammatory cells recruited to the injured vessel [20]. The protective effect of NCX 4016 was in part due to the vasoactive action of nitric oxide (NO) released from the molecule, since the dose of ASA used had the equivalent ASA concentration as the NCX 4016 drug dose used. NO is also involved in the control of several pathophysiological responses, including inflammatory cell adhesion, vascular reactivity, endothelial permeability and regulation of smooth muscle cell proliferation [23], [24].

In the aortic arch we saw a decrease in the number of initial lesions in mice treated with high dose ASA compared with age-matched controls, but the total plaque area was increased after a 30 week follow-up. This is consistent with a previous report where low dose ASA inhibited the initiation of lesions in the artery of ApoE^−/−^ mice on a high cholesterol diet, but long term administration eventually stimulated lesion growth [25]. In our study ASA decreased the collagen content of advanced BCA lesions of the aortic arch in unirradiated mice. This contrasts with a study in LDLR^−/−^ mice that showed that aspirin significantly reduced vascular inflammation in aortic atherosclerotic lesions of unirradiated mice and increased the amount of collagen in the atherosclerotic plaques, thereby increasing plaque stability [26].

### Anti-inflammatory drugs and endothelial cell damage

Radiation has been shown to upregulate endothelial cell adhesion molecules, such as ICAM-1, VCAM-1 and P-selectin, especially in microvascular or tumor endothelial cells [11], [12]. High dose ASA reduced the expression of VCAM-1 in irradiated carotid arteries, however neither NCX 4016 nor ASA had any significant effect on ICAM-1 expression in our studies. Previously published in vitro studies have shown that ASA is able to inhibit induction of VCAM-1 in TNF stimulated HUVECs [27]. However, in vivo studies in arteries of hypercholesterolemic rabbits or ApoE^−/−^ mice showed either no change in expression of VCAM-1, or only a small reduction [25], [28]. No previous publications have reported on the effects of anti-inflammatory drugs in irradiated arteries of hypercholesterolemic animals.

Thrombomodulin controls the balance between the pro-coagulant activities of thrombin (fibrin generation, platelet activation) and anticoagulant activity, and determines a condition of either normal homeostasis (high expression of TM) or vessel pathology (low expression of TM). Clinical studies and experiments in animal models have demonstrated that exposure of normal tissue to ionizing radiation is associated with rapid and sustained loss of thrombomodulin in microvascular endothelial cells [29], [30]. In our model of irradiated carotid arteries, a reduction of thrombomodulin expression around the bifurcation was seen after treatment with high dose ASA. This might be expected to counteract the ASA-induced reduction in VCAM-1 expression. NCX 4016 did not have this effect on thrombomodulin.

Overall, we conclude from the immunohistochemical stainings that neither NCX 4016 nor high dose ASA shifted the balance of pro- and anti-inflammatory/thrombotic molecules at 4 weeks after irradiation.

### Anti-inflammatory drugs and radiation-induced atherosclerosis

In previous studies, we have shown that irradiation of the carotid arteries of ApoE^−/−^ mice accelerated the development of atherosclerosis and predisposed to the formation of an inflammatory, thrombotic plaque phenotype [7], [8]. However, the effect of irradiation on the aortic arch is different compared to the carotid lesions. Although we see a similar (two-fold) increase in the number of initial lesions in both the aortic arch region and carotid arteries, plaque size of the BCA lesion was strikingly smaller in the irradiated animals. Vascular site-specific effects on the development of atherosclerosis is a frequently reported phenomenon [31]. In the context of irradiation, Schiller and colleagues also reported site-specific differences in lesion development after 10 Gy whole body irradiation and subsequent bone marrow transplantation (BMT). In this study, thoracic aortic lesion area decreased, while lesion area in the aortic valve region increased after BMT [32]. It is assumed that variation in lesion development at different sites is sensitive to several parameters, such as genetic background, immune status, gender, hemodynamic factors and differences in endothelial or smooth muscle cell gene expression [31], [33]. In the current study, site specificity was observed in both the effect of the intervention (protecting effect of NCX 4016 in carotid arteries of unirradiated animals, while ASA stabilized plaque development in the irradiated animals) and the effect of irradiation itself in the carotid and aortic arch region. The underlying mechanisms remain to be determined.

### Conclusions

Although modest benefits were obtained by NCX 4016 in the unirradiated animals, in terms of inhibition of carotid lesions, the drug was not effective at inhibiting lesion development in the irradiated animals. Irradiation could be too strong a challenge to be counteracted by NO. Irradiation causes chronic oxidative stress [34] which may reduce the protective effect of pharmacologically released NO. The effects of ASA on development of atherosclerosis were site-dependent. ASA reduced the number of initial lesions in the unirradiated aortic arch but increased the total plaque burden. The collagen content of advanced carotid lesions increased in irradiated mice treated with high dose ASA compared to mice treated with control chow, indicating that ASA “stabilized” the plaques reversed the radiation-induced reduction in collagen seen in both the current study and our previous studies [7], [8]. However, this effect was not seen in the BCA.

The effects of radiation-induced atherosclerosis could not be circumvented by these anti-inflammatory and anti-thrombotic therapies, suggesting other underlying mechanistic pathways compared to age-related atherosclerosis.

## Materials and Methods

### Ethics statement

Mice were housed according to guidelines of the Netherlands Cancer Institute, and procedures were carried out in compliance with standards for use of laboratory animals. Animal experiments performed in this manuscript have been approved by the animal experimental committee of the Netherlands Cancer Institute (DEC-consult).

### Experimental design and irradiation

ApoE^−/−^ mice on a C57BL/6J background were bred in isolator cages at the breeding facility of the Netherlands Cancer Institute. After weaning, at 3 weeks of age, male mice were housed in filter-top cages and given free access to standardized mouse chow (3.7% fat, RM1 (E) SQC, SDS, London, U.K.) and acidified drinking water. NCX 4016 (3-(nitrooxymethyl)phenyl 2-(acetyloxy) benzoate) was provided by NicOx SA, France and ASA was purchased from Sigma. At the age of 9–10 weeks the mice were given NCX 4016 (555 mg/kg food) or ASA at low or high doses (278 or 2,778 mg/kg food) in the chow. Control mice received the same chow without any drugs. The food consumption was recorded throughout the follow-up period and used to estimate the daily drug intake per mouse. This indicated daily doses of >60 mg/kg NCX 4016, >30 mg/kg ASA or >300 mg/kg ASA. The final doses of ASA and NCX 4016 were based on published studies in mice and rats on cardiovascular endpoints [22], [35]–[37]. The mice received the chow one week before irradiation and continuously throughout the experiment. For the short term experiments (4 week follow-up) all mice received 14 Gy irradiation to the neck region and for the long term experiments half of the mice received 14 Gy irradiation and the other half received sham irradiation (0 Gy) and the mice were sacrificed 30 weeks after irradiation.

Irradiation was in a dorso-ventral direction, with 250-kV X-rays, operating at 12 mA and filtered with 0.6 mm of copper. The field size was 20×15 mm, encompassing both carotid arteries, the aortic arch, and basal portion of the heart, but with the lung outside the 100% iso-dose region. The mice had a body weight of 20-30 g to ensure correct positioning of the carotid arteries within the irradiation field.

### Platelet aggregation

In half of the mice per group, platelet aggregation was measured immediately prior to sacrifice, using a two-channel whole-blood aggregometer (CH 590-2D with Aggrolink, Chronolog Corp., Havertown, PA). Blood samples were collected via heart puncture under anesthesia and diluted with 0.38% citrate buffer (9:1 v/v). All analyses were performed within 25 min from blood sampling. A 500 µl sample of citrated blood was added to the aggregometer cuvette containing 500 µl saline and stirred at 37°C. Arachidonic acid (Chonolog, final concentration 0.1 mM) was used to stimulate aggregation. Changes in electrical impedance were monitored for 15 min and quantified by reference to a standard internal resistance.

### Blood sampling and analysis

Half of the mice were sacrificed after overnight fasting, and blood samples were taken by cardiac aspiration. Blood samples were collected in EDTA tubes (Becton Dickinson, Alphen aan den Rijn, the Netherlands) and centrifuged at 3500 rpm for 15 minutes at 4°C. Plasma samples were frozen at −70°C. Plasma cholesterol and triglyceride levels were determined by standard colorimetric enzyme assays (Cobas integra 400, Roche Diagnostics, Almere, The Netherlands).

### Tissue handling

#### Short-term experiments (4 weeks after irradiation)

Directly after sacrifice and blood sampling, the arterial system was perfused with 0.1 mg/ml sodium-nitroprusside in phosphate-buffered saline (PBS) under standard pressure (100 mm Hg) for 3 minutes. The carotid arteries were excised, stained with eosine and separately embedded between 2 pieces of liver tissue to provide rigidity for the fragile arteries during sectioning. The preparation was rapidly frozen on dry ice and stored at −70°C.

#### Long-term experiments (30 weeks after irradiation)

Directly after sacrifice and blood sampling, the arterial system was perfused with 0.1 mg/ml sodium-nitroprusside in PBS under standard pressure (100 mm Hg) for 3 minutes, followed by 1% paraformaldehyde fixative (3 minutes). The cervical, thoracic and abdominal arterial tree and the heart were excised and the entire preparation was attached to a cork sheet and fixed for 24 hours in 1% paraformaldehyde before transfer to 70% alcohol. The lungs were excised separately. The aortic arch, carotid arteries, descending thoracic aorta, renal arteries, heart and lungs were embedded in paraffin.

### Morphometric analysis of plaque

#### Short-term experiments (cryostat sections)

Serial cross-sections of 7 µm were collected on 20 glass microscope slides, which gave 140 µm between adjacent cryostat sections on one slide as described before [8]. One slide was stained with hematoxylin and eosin (H&E) and the section at the site of the bifurcation (position 0) and one section above (position 1) and below the bifurcation (position -1) was scored to identify the presence of fatty streaks.

#### Long-term experiments (paraffin embedded arteries)

For the quantitative analysis of plaque area and number, approximately 35 longitudinal, serial sections of 4 µm were made from each carotid artery and the aortic arch. Every fifth section (20 µm apart) was stained with H&E. Plaque area was measured on four of these sections artery, selected to cover the central part of the lesion, and the average of these measurements was recorded per lesion. Total numbers and areas of lesions in left and right carotid artery were calculated per mouse. In the aortic arch the total number of lesions was determined in the complete aortic arch and the plaque area was measured only in the brachiocephalic artery (BCA). All plaques were categorized as initial lesion (macrophage rich, without a thick fibrous cap) or advanced (well-defined, necrotic/lipid core or thick fibrous cap), using a modified American Heart Association classification scheme [38], [39]. The sections were also examined for the presence of collagen (Sirius Red staining), fibrin (Martius-scarlet-blue trichrome staining), iron-containing macrophages (Perl staining) and granulocytes (H&E and confirmed with a Ly6G staining). Morphometric parameters (plaque area and collagen content) were analyzed using a microscope coupled to a computerized morphometry system (Leica Qwin V3, Leica, The Netherlands). The relative collagen content was determined by dividing the positive collagen area by the individual plaque area. All measurements were made, without knowledge of the treatment group, by one investigator.

### Immunohistochemistry

Cryostat sections of the carotid arteries were air-dried and the slides were fixed for 10 min in acetone at −20°C (except for CD31). Endogenous peroxidase activity was blocked with 0.3% hydrogen peroxide in methanol for 30 min. Endogenous biotin was blocked with the avidin/biotin blocking kit (DAKO, ITK Diagnostics BV, Uithoorn, The Netherlands). After blocking of nonspecific binding sites with protein block serum free (DAKO), slides were incubated overnight with primary antibodies at 4°C. After rinsing with PBS, the slides were incubated with the biotinylated secondary antibodies rabbit anti-rat IgG (45 min), rabbit anti-hamster IgG (45 min) or swine anti-rabbit IgG (30 min). All antibodies were purchased from DAKO. Antibody reactivity was detected using HRP (horse radish peroxidase)-conjugated biotinavidin complexes (Vector Laboratories, Burlingame, CA) and developed with DAB solution (Sigma). Primary antibodies comprised: rat anti-mouse CD31 (Platelet Endothelial Cell Adhesion Molecule, PECAM-1, Becton Dickinson, Alphen aan de Rijn, The Netherlands) diluted 1∶50, hamster anti-mouse CD54 (ICAM-1, Becton Dickinson) diluted 1∶50, rat anti-mouse CD106 (VCAM-1, Becton Dickinson) diluted 1∶25, rabbit anti-mouse thrombomodulin (American Diagnostica, Greenwich, CT) diluted 1∶200 and rabbit anti-mouse eNOS (endothelial nitric oxide synthase, Santa Cruz Tech., Santa Cruz, CA) diluted 1∶200. Primary and secondary antibodies were diluted in 1% BSA/PBS. As a control for background staining, control slides were treated in the same manner, except 1% BSA/PBS was substituted for the primary antibodies. Within one experiment all sections were processed identically, at the same time with precisely the same incubation times for the primary and secondary antibody and DAB. Therefore, all differences between the treatments are ultimately due to DAB identification of the relevant protein.

### Quantitative analysis of the immunohistochemistry

The amount and intensity of CD31, ICAM-1, VCAM-1, thrombomodulin and eNOS staining in the endothelium of the carotid arteries at three positions around the bifurcation (−1, 0 and 1) were quantitatively analyzed. Each tissue section was photographed and analyzed using a microscope (20× objective) coupled to a computerized morphometry system (Leica Qwin V3, Leica). The endothelial cell layer was determined as representing the inner most layer of the vessel wall automatically detected from the lumen contour. The immunopositive signal in the endothelial cell layer was measured by setting a threshold on the RGB monochromatic channels as determined by the observer. The threshold values of each staining were then held constant in the analysis of all images. The relative immunopositive staining was calculated as the sum of the immunopositive area divided by the sum of the total endothelial area (area(x)). The area of CD31 expression was considered as a total area of intact endothelium (area(CD31)). The average optical density (OD) of these areas was automatically calculated (0–255**:** 0 =  dense amount and 255 =  none). The area of ICAM-1, VCAM-1, thrombomodulin and eNOS expression indicates the percentage of activated endothelial cells calculated as: area(x)/area (CD31) x 100%.

### Statistics

Quantitative morphometric analyses of plaques and quantitative immunohistochemical analysis were expressed as means ± SEM and irradiated and unirradiated groups were compared using a non-parametric Mann-Whitney U-test. Data for assessment of fatty streak formation, thrombotic and inflammatory plaque characteristics were expressed as incidence of carotid arteries containing lesions with these phenotypes, and the groups were compared using the Fisher's Exact test. Group differences were considered statistically significant at p<0.05.
